# Prospective nonrandomized observational study of the use of an impedance threshold device in patients with spontaneous respiration and hemodynamic instability

**DOI:** 10.1186/cc14224

**Published:** 2015-03-16

**Authors:** C Pantazopoulos, I Floros, N Archontoulis, D Xanthis, D Barouxis, N Iacovidou, T Xanthos

**Affiliations:** 1 Laiko General Hospital of Athens, Greece; 2 University of Athens, Medical School, Athens, Greece

## Introduction

The use of an impedance threshold device (ITD) in cardiac arrest victims has been shown to increase the systolic arterial pressure (SAP) by increasing venous return [1]. There are limited studies concerning the use of ITD in patients with spontaneous respiration and hemodynamic instability. The purpose of this study is to evaluate changes of hemodynamic parameters with the use of ITD in patients with spontaneous respiration and hemodynamic instability.

## Methods

A 5-month prospective nonrandomized observational study that included 50 adult patients with spontaneous respiration and hypotension in the emergency room and the wards of multiple causes except trauma. After measurement of the SAP and verification of hypotension (SA*P *≤90 mmHg), a mask-style ITD was added. Hemodynamic parameters were evaluated every 1 minute and for 10 minutes after the intervention. Endpoint of the study was a change of patient's SAP after application of ITD.

## Results

The SAP of patients that were included in the study increased 15 to 22 mmHg (*P *< 0.05). Heart rate remained unchanged. Eighty percent of patients declared good to very good tolerance from ITD application.

## Conclusion

In this observational study of patients with spontaneous respiration and hypotension, ITD increased the SAP, while it seems that it was well tolerated by patients. Additional and larger studies will be needed in the future in order to investigate the benefits of ITD when used to patients with spontaneous respiration and hemodynamic instability.
